# Observations on the Treatment of the Venereal Disease without Mercury

**Published:** 1818-06

**Authors:** 


### *Observations on the Treatment of the Venereal Disease without Mercury.* By G. J. Guthrie, Esq.

( Medico-Chirurgical Transactions, vol. viii. )

Of all the papers which we have read on this interesting subject, Mr. Guthrie's appears to us the most judicious, and the most reasonable. From so respectable an authority we shall differ with caution; but we are convinced that Mr. Guthrie has too much liberality of sentiment, and too much zeal in the cause of science, not to wish for rather than prevent a calm and dignified investigation of so momentous a point in therapeutics, as that under consideration. We shall therefore enter into the analysis of this document with a most anxious desire to elicit truth, and with the most perfect abhorrence of every thing in the shape of quibbling or criticism.

Mr. Guthrie observes, that on the continent, in general, little *attention* is paid to the *appearance* of primary sores.

" If a man have had a suspicious connection, followed by ulcers on the glans penis, or prepuce, or even a gonorrhœa, he is at once declared to be infected with the venereal disease; but this does *not* lead, in general, in Italy, or in the south of Europe, to the exhibition of *mercury*, or any other specific. In France, and particularly in Paris, the *contrary* is the general practice; the patient is placed on the oxymuriate of mercury, and after taking about thirty-two *portions* in half doses, twice in the day, which generally occupy the same number of days, he is considered free from disease, and this will, in most cases, be true *in*

*all kind of sores which have originated from sexual intercourse;* but if the ulcers should not heal in this time, or secondary symptoms supervene, he frequently continues the medicine for an unlimited time. In doing this, the Parisian surgeons are, however, acquainted with a fact, which has, until very lately, been denied in England, viz. that every kind of ulcer is *curable* by common means; and M. Cullerier, the first surgeon in the venereal hospital at Paris, demonstrates the possibility of doing so every year to his class; *but after the ulcers are healed, he puts each patient through the usual course, to prevent secondary symptoms.*" 552.

Upon this passage we would observe, that the fact of the spontaneous *disappearance* (for we do not call it *cure*) of primary ulcers has been long known to every practitioner of observation in this country, and more especially to the naval surgeons in the late war, from a circumstance which is related in the first article of this Journal.* Again, considering the indiscriminate administration of *mercury* in France, in all kinds of primary and secondary symptoms, and the almost total neglect of that remedy in Portugal, for instance, it becomes extremely desirable to ascertain the comparative *results* of these two different modes of practice. We have been for some years past in the habit of dipping a good deal into French medical literature; but have not met with any thing worthy of notice, on the inutility or the injurious effects of mercury in venereal diseases; and indeed Mr. Guthrie's own words prove this to be true; for if the first surgeon of the venereal hospital at Paris be not considered a competent judge on this point, we know not to whom we can appeal. Lagneau, the latest writer on the complaint in question, inculcates the use of mercury even in gonorrhœa. In respect to Portugal, we shall quote a passage from Mr. Guthrie himself.

"If (says he) we refer to Dr. Fergusson's paper, we shall find it stated, that, in *many cases* in which the Portuguese certainly give no mercury for the cure of primary symptoms, the secondary ones run their usual course, even to the loss of the bones of the nose; and I am very willing to confirm a remark he once made to me, that there were more people to be met with in the town of Lisbon, who had suffered that mutilation, than in any other he has seen of the same size." 562.

Granting the *possibility* of curing syphilis without mercury, this *chance* of mutilation is a weighty consideration!

---

* Vide p. 445.

Mr. Guthrie states, that, between the years 1801 and 1809, he was surgeon of the 29th regiment, and had great experience in the disease under consideration.

"In the period to which I have alluded (says he) a great number of persons afflicted with this disease came under my care; they nearly all underwent a moderate course of mercury, provided the ulcers did not assume a healing appearance at the end of a fortnight or three weeks, and *I very seldom* had a case of secondary syphilis, not even among those who were occasionally from under my inspection. *I am not aware of having ever discharged or lost a man in consequence of it.*"

This is surely strong evidence on the point under discussion. But Mr. Guthrie goes still farther. He says, that as later experience has proved that a great number of these patients would not have had secondary symptoms if mercury had *not* been exhibited, it follows that it was *uselessly* exhibited, and yet did no injury to the constitution, and consequently that "it is not the cause of all the evil which, in many cases, is attributed to it." And again,

"It appears to me, that it is only where mercury is persisted in after it has evidently ceased to do good; when it disagrees with the constitution, or when it is exhibited at an improper period, or very irregularly, the patient being exposed to wet and cold, that it produces those symptoms usually supposed to depend upon it. The fact I have stated, as to the non-occurrence of secondary symptoms in regimental hospitals, *where all doubtful cases were treated by mercury*, is so positive, that I am certain no regimental surgeon of ability will be found to contradict it. In the half year ending the 24th of June, near 1400 cases of primary symptoms were treated in the army in France by mercury, and in this period only 14 cases of secondary symptoms occurred." 568—9.

Now, where it is acknowledged that one case in ten is followed by constitutional symptoms, where no mercury is used, whereas this document proves that mercury secures ninety-nine out of a hundred, it is surely madness to adopt a new practice which gives only *one-tenth* the security of the old! It is moreover stated by Mr. Guthrie, that ulcers resembling the true venereal chancre, required from six, eight, to ten, twenty, and in one case twenty-six weeks for cure!

"I have every reason (says this experienced surgeon) to be certain that almost all these protracted cases would have been cured in one-half, or even one third of the time, if a moderate course of mercury had been resorted to." 559.

Finally, Mr. G. gives this memorable lesson to the practitioners of the new light.

"But I again repeat, if any one should be disposed to try the method of cure, the effects of which I have noticed, let him constantly bear in mind, that every case so treated requires as much attention and quietude on the part of the patient, *and more attention and discrimination* on the part of the surgeon, than where mercury is used for the cure." 580.

Under every view of the case then, we conceive that the new plan of treating *syphilitic* affections is most hazardous, cruel, and—we had almost said—unjustifiable. It is so hazardous to the reputation of the surgeon, as well as to the health of the patient, that we are convinced it never will be generally adopted in private practice, until a total revolution takes place in the nature of the disease, of which Cytherean milennium there does not appear much hope at the present moment. We think the plan unjustifiable, *if* continued an instant after its *comparative* demerits are proved; and proved they are by the foregoing document, or all human testimony is vain. The end and object of medical science is the *mitigation* of human afflictions. No pathological investigation can authorize us to depart from this principle in any shape, or on any occasion. But although we condemn a perseverance in this new mode of treatment, we are ready to allow, with the intelligent author of the paper under consideration, that advantage may be taken of the facts already stated, and often turned to the essential benefit of the patient. In scrofulous subjects, who unhappily contract ulcers resembling chancres, and to whom a course of mercury might be injurious (according to received opinions), it must be satisfactory to know that these ulcers will heal by simple means;

"—and that if they be regular in their mode of life, secondary symptoms *may not* follow; or if they should, that there is still a *probability* of their being cured without the use of that remedy, which, to these people, may prove a greater scourge than the disease for which it is administered." 579.

After all, the investigation revolves to the same point from whence it started, namely, the *discrimination* of syphilitic from syphiloid affections. To this path we must at present confine ourselves.

